# Genetic Polymorphism Drives Susceptibility Between Bacteria and Bacteriophages

**DOI:** 10.3389/fmicb.2021.627897

**Published:** 2021-03-24

**Authors:** Xiaoxu Zhang, Dongyan Xiong, Junping Yu, Hang Yang, Ping He, Hongping Wei

**Affiliations:** ^1^Key Laboratory of Emerging Pathogens and Biosafety, Centre for Biosafety Mega-Science, Wuhan Institute of Virology, Chinese Academy of Sciences, Wuhan, China; ^2^College of Life Science, University of Chinese Academy of Sciences, Beijing, China

**Keywords:** bacteriophage, *Staphylococcus aureus*, experimental evolution, genetic polymorphism, minor alleles

## Abstract

Phage therapy has attracted much attention for the treatment of antibiotic-resistant bacteria in recent years. However, it is common for bacteria to obtain resistance capability in short time after interaction with a lytic phage, as observed in phage therapy and co-culture of host and phage in a lab. In order to understand the mechanisms behind resistance, *Staphylococcus aureus* AB91118 and its lytic phage LQ7 were studied as a model system. A mutant strain named R1-3-1 resistant to the ancestral phage LQ7 was isolated, and then phages experimentally evolved from LQ7 were able to kill R1-3-1. Genomes of the two bacterial strains and the three phages (LQ7, ELQ7P-10, and ELQ7P-20) were analyzed based on deep sequencing data of NGS. Analyses showed that a few mutations could be identified in R1-3-1 and the evolved phages. Instead, in all the genomes of the bacteria and the phages, there exists genetic polymorphism of minor alleles, which distributes in many functional genes. Specifically, in the AB91118-LQ7 system it was found that the unique polymorphism sites in R1-3-1 associated to metabolic pathways could be inhibited by chloramphenicol (CHL). The resistant mutant R1-3-1 could become sensitive to the phage LQ7 in the presence of CHL. Combined use of CHL and the evolved phage from 20 cycles (ELQ7P-20) could produce the least resistance when killing the bacteria AB91118. The genetic polymorphism of minor alleles would be a new mechanism to drive the co-evolution between a phage and its host, which may enable the phage and the host get ready and fast response to the selective pressure from one to the other.

## Introduction

With the emergence of antibiotic-resistant bacteria such as methicillin-resistant *S. aureus* (MRSA) and vancomycin-resistant *S. aureus* (VRSA), the treatment of bacterial infections through antibiotics has become increasingly problematic (Sakoulas et al., 2005; Senok et al., 2019). As an alternative, phage therapy has attracted new interest for the treatment of antibiotic-resistant bacteria in recent years (Levin and Bull, 2004; Lyon, 2017). Normally lytic bacteriophages were used for phage therapy (Sulakvelidze et al., 2001). For example, phage therapy has been found to be effective for treatment of staphylococcal infections in animals (Garcia et al., 2019; Lehman et al., 2019). Clinical trials in human (Kishor et al., 2016; Ooi et al., 2019; Prazak et al., 2019) are also in progress. However, like antibiotic resistance, the use of phages can lead to the emergence of phage-resistant strains, which would be a significant obstacle for phage therapy (Denes et al., 2015; El Haddad et al., 2018). It is important to understand how bacteria and bacteriophage evolve in the presence of the other.

Previous studies have shown that the interaction between a phage and a bacterium is a co-evolutionary arms race. Bacteria could resist the infections of phages by a range of antiviral mechanisms such as adsorption-blocking systems, blocking phage DNA entry, restriction–modification (R/E) systems, CRISPR Cas systems, and abortive infection (Abi), etc (Labrie et al., 2010; Safari et al., 2020). Phages, in response, can evolve multiple tactics to overcome these defensive strategies in order to thrive in most environments (Labrie et al., 2010). For example, phages can evolve by specifically modifying their genomes to gain increased capability such as a broader host range, a faster adsorption or a more effective bactericidal capacity to defeat bacterial defense (Samson et al., 2013). Coevolution between bacteria and bacteriophages can be characterized as an infinitive constant evolutionary battle (phage-host arms race) (Golais et al., 2013). However, most of the current studies have mainly focused on the mutations or indels of genes in bacterial and phage genomes. For example, Denes et al. (2015) revealed that mutations associated with phage resistance in *Listeria monocytogenes* were found primarily in two loci, which link to phage adsorption. The phage resistant mutants of *Acinetobacter baumannii* had a single-nucleotide deletion resulting in a frameshift in a gene within the K locus (Gordillo Altamirano et al., 2021). Another lytic phage SPO1 could gain a broader host range against *Bacillus subtilis* after having mutations in two genes, encoding the baseplate, and the fiber proteins required for host attachment (Habusha et al., 2019). It needs more studies to understand how these mutations were generated. It is also not clear if a phage could evolve further to obtain the capability to kill the phage-resistant bacteria. Finding the mechanisms behind will be important not only to understand the co-evolution between host and phage, but also to find the solutions to overcome phage resistance during phage therapy.

In present study, we try to understand how bacteria evolve resistance in the presence of a lytic phage, which is normally chosen for phage therapy, and if the phage could evolve further experimentally to overcome the developed resistant bacteria. *Staphylococcus aureus* AB91118 strain and its novel lytic dsDNA phage LQ7, a natural isolate from a raw river sample (Yan et al., 2017), were studied as an example. The results showed that besides mutations, a new mechanism, minor alleles in the genomes of the bacteria and the phages, would be important factors contributing to the resistance of the host to the phage and vice versa.

## Materials and Methods

**FIGURE 1:**
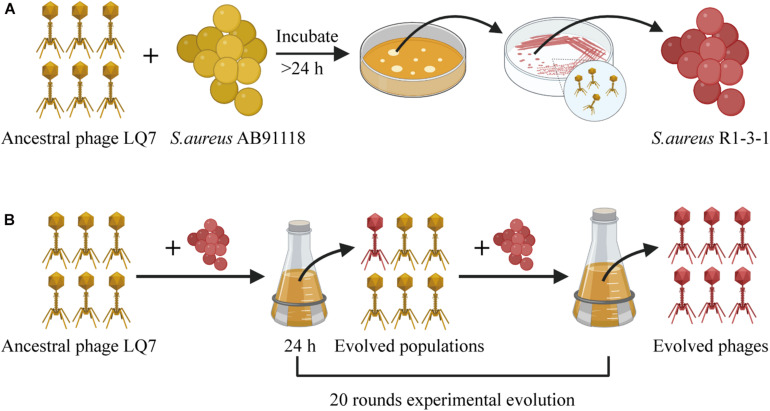
The overall experiment design for studying the evolution between *S. aureus* AB91118 and lytic phage LQ7. **(A)** Phage-resistant mutant isolation. **(B)** Experimental evolution of phages.

The design for the overall experiment is shown in Figure 1. Briefly, *S. aureus* AB91118 was cultured with a lytic phage LQ7 for more than 24 h on an agar plate and then a colony named R1-3-1 resistant to the phage LQ7 were isolated. After that, LQ7 were experimentally evolved with R1-3-1 for 20 rounds. Genomes of *S. aureus* AB91118 and mutant R1-3-1 were sequenced and analyzed to find their difference. Genome sequences of LQ7 and the evolved phages at 10th and 20th round were also analyzed and compared with each other to find the possible changes in their genomes.

### Strains and Culture Conditions

*Staphylococcus aureus* AB91118 was bought from China Microbe Collection Center (CMCC) and cultured at 37^o^C with shaking (180 rpm) in Luria-Bertani (LB) broth. Phage LQ7 was previously isolated from a raw river sample (Yan et al., 2017). Plaque purification and enumeration of phage titers were all performed using the double-layer overlay method (0.7% agar for the top layer and 1.5% agar for the LB layer).

### Isolation of Phage-Resistant Bacterial Mutant R1-3-1

Isolation of a phage-resistant mutant was performed as described in previous studies (Habusha et al., 2019). Briefly, a single colony of *S. aureus* AB91118 formed on agar plate was inoculated into LB broth and cultivated at 37^o^C with shaking at 180 rpm. After cultivation for 8 h to exponential growth phase, the suspension containing 5 × 10^8^ CFU/mL (OD_600_ ≈ 0.45) of *S. aureus* AB91118 was mixed with phage LQ7 at a multiplicity of infection (MOI) of 100. The mixture was left at 37^o^C for 10 min for the adsorption and infection of the phage. Then the infected culture was poured on a double-layer plate and incubated until form of some phage-resistant bacterial colonies (in about 24 h) as shown in Figure 1A. After that, a single colony was picked up and inoculated onto a phage containing semisolid plate for further culture to obtain resistant mutants. After repeating this process for five times, a phage-resistant mutant named as R1-3-1 was isolated.

### Growth Rate of Bacteria

Growth rates of *S. aureus* AB91118 and R1-3-1 were measured by adding 200 μL of the bacteria solution in fresh LB medium (OD_600_ ≈ 0.45) into a 96-well plate for 10 h using a microplate reader (Biotek, US), which tracks optical density at 600 nm (OD_600_). The growth rates were also measured in the presence of phage LQ7 at MOI ≈ 10. At the same time, 200 μL of LB liquid medium without bacteria and phage buffer (50 mM Tris-Cl, pH 7.5, containing 150 mM NaCl, 10 mM MgCl_2_⋅6H_2_O and 2 mM CaCl_2_) were used as blank controls. The experiments were performed in triplicate.

### The Experimental Evolution of Phage LQ7

The experimental evolution of phage LQ7 was conducted as reported previously (Akusobi et al., 2018). Briefly, phage-resistance bacterial mutant R1-3-1 at stationary-phase were stored at −80^o^C to keep them stable in aliquots and one aliquot was used for each round of the phage evolution experiment. At the start of the experiment, 20 μL of ancestral phage LQ7 (1 × 10^9^ PFU/mL) and 200 μL of the stationary-phase R1-3-1 were added to a conical flask containing 20 mL LB. The mixture was incubated at 37^o^C for 24 h with shaking. After that, the mixture was centrifuged with 16,000 × *g* for 10 min at 4^o^C. Then the lysate in the supernatant was filtered through a 0.22 μm sterile filter. 20 μL of the filtered lysate was mixed with 200 μL of the stationary-phase bacteria R1-3-1 again to perform the next round. This process was repeated for 20 successive rounds. The lysate of each round, containing evolved phages, was collected and stored at 4^o^C until use.

## Characterization of Ancestral Phage LQ7 and Evolved Phages

### Infection Activity Test

A total of 500 μL of R1-3-1 in exponential growth phase with a concentration of 5 × 10^8^ CFU/mL was mixed with melted semisolid agar (0.7% top agar) and poured onto solid Luria Bertani agar plate first. Then 2.5 μL of ancestral phage LQ7 and the evolved phages were dripped into the soft agar containing R1-3-1, respectively. After overnight incubation, spot formation was examined.

### Effects on Bacterial Growth

Growth of R1-3-1 infected with either the ancestral phage LQ7 or the evolved phages from different rounds of evolution (ELQ7P-5, ELQ7P-10, ELQ7P-15, and ELQ7P-20) were measured in a 96 well plate as described above. Each well was added with 100 μL LB medium, 50 μL bacteria (5 × 10^8^ CFU/mL) and 50 μL phage (5 × 10^8^ PFU/mL) at MOI = 1. LB medium was used as the blank control. The experiments were performed in triplicate.

### Transmission Electron Microscope (TEM)

The phage LQ7 and four evolved phages (ELQ7P-5, ELQ7P-10, ELQ7P-15, and ELQ7P-20) was analyzed using transmission electron microscopy (TEM). Briefly, the amplified phages were concentrated at 4^o^C using CsCl density gradient ultracentrifugation at 2,10,000 × *g* for 2 h in a Beckman SW 41 Ti rotor. The phage collected was dialyzed using a cellulose membrane in phage buffer for 12 h. Then 20 μL of the phage suspension was dripped on a carbon-coated copper grid and allowed to adsorb for 10 min. Excess liquid was drawn off carefully by touching the side of the grid with filter paper. The grid was allowed to dry in air. Phages were negatively stained with freshly prepared 2% (wt/vol) phosphor tungstic acid (PTA) for 3 min. The copper grids were air dried for 2 h and then observed under a transmission electron microscope (Hitachi H-7000FA, Japan) at an operating voltage of 75 kV.

### Adsorption Assay

Adsorption rate of phages to bacteria was determined as described previously (Uchiyama et al., 2017). Mixture containing 200 μL of bacteria (OD_600_ = 0.6), 100 μL of phage (1 × 10^4^ PFU/mL) and 200 μL of LB medium was incubated for 7.5 min at 37^o^C. Respective phages in LB medium were used as the control groups. After pelleting the bacterial cells by centrifugation (21,000 × *g*, 1 min), the concentration of the unbound phages in the supernatant was measured using a plaque assay. The experiments were performed in triplicate.

## Genome Sequencing and Analysis

### DNA Extraction and Sequencing

To extract DNA from *S. aureus*, *S. aureus* cells were harvested by centrifuging 5 mL of bacteria in exponential growth phase at 14,000 × *g* for 5 min. The pellets were re-suspended in 500 μL TE buffer (10 mM Tris–HCl, 1 mM EDTA, pH 7.6) and lysed with lysostaphin (1mg/mL, Sigma-Aldrich, Saint-Louis, MO, United States) at 37^o^C for 1 h. The cell lysate was then treated with 5 μL RNase A (10 mg/mL) (Thermo Fisher Scientific) at 37^o^C for 1 h to remove RNA, followed by adding 0.5 mg/mL proteinase-K and incubation at 56^o^C for 1 h. Finally, bacteria genomic DNA was extracted using the phenol-chloroform protocol as described previously (Sun, 2010).

To extract phage DNA, a single plaque of LQ7 were picked and propagated to obtain 20 mL of phage lysate for the final DNA preparation. Genomic DNA of the evolved phages at round 10th and 20th (ELQ7P-10 and ELQ7P-20) were extracted from 20 mL of the lysates directly (Kering et al., 2020). The quality and quantity of the DNA obtained were determined using a NanoDrop spectrophotometer (ND-2000, Thermo Fisher Scientific, Waltham, MA, United States). Genome high-throughput sequencing was done by Benagen Inc (Wuhan, Hubei, China) using Illumina NovaSeq 6000 sequencer for the phages and the bacteria and 150 bp pair-end reads were generated. Furthermore, Nanopore PromethION platform was performed on the machine of Oxford Nanopore Technologies, ONT for the bacteria.

### Variation and Polymorphism Analyses of Bacteria and Phage Genomes

The bacterial genome sequences were obtained by the Nanopore platform sequencing data using software Wtdbg2 and Minimap (Li, 2016; Ruan and Li, 2020) and errors were corrected using the high quality NGS data (> Q30) on the base-level through Nanopolish software (Loman et al., 2015). The phage LQ7 genome was *de novo* assembled from the NGS data by SPAdes v3.13.0 (Bankevich et al., 2012) with the default parameters. During the genome assembling, each base with major allele frequency was also analyzed. In addition, the software Prokka^1^ was used to annotate the bacteria and phage genes.

For the genetic variation analysis of the bacteria and the phages, the clean reads of the NGS data were aligned to the reference genomes (AB91118 genome for the bacteria and LQ7 phage genome for the phages) using BWA software (Li and Durbin, 2009). The sequencing depth was calculated by the aligned reads using SAMtools and Bedtools (Li et al., 2009; Quinlan, 2014). Mutation information was archived by software Bcftools (Narasimhan et al., 2016).

For the genetic polymorphism analysis of the bacteria and the phages, the sequencing error rates of NGS were estimated using Jellyfish software with the default options (Marçais and Kingsford, 2011). The genetic polymorphisms were identified based on the minor allele frequency (MAF). The minor allele frequency (MAF) was calculated by the VarScan software (Koboldt et al., 2012) (version: 2.3.9, with the default options chose) and confirmed by the IGV software (Thorvaldsdóttir et al., 2013) (version 2.7.2). According to the sequencing error rates and related studies (Broniewski et al., 2020), the sites with the both minor allele frequency (MAF) greater than 0.03 and sequencing depth at least 100 were considered as polymorphism sites to minimize sequencing errors and noises. This approach may inevitably underestimate the true number of the polymorphism sites since the sites with lower MAF were filtered out to limit the noise caused by sequencing errors.

Finally, genes with polymorphism sites as well as mutations in the genomes were identified by a R script. Five identified polymorphism sites with higher MAF and the mutations were verified by PCR and Sanger sequencing using the designed primers based on the genome sequences. Gene Ontology (GO) (Gene Ontology Consortium, 2015) enrichment analysis was performed through the R package ClusterProfiler; Yu et al. (2012) with default options based on the genes with the polymorphism sites that occurred only in the R1-3-1 genome. All statistical analysis was performed by the R program (version 3.5.3)^2^.

## Effects of Bacteriostatic Antibiotics and Phages on the Growth of R1-3-1 and Mutant Frequency of AB91118

### Minimum Inhibitory Concentration (MIC) of Chloramphenicol

The stock solution of chloramphenicol (CHL) (Sigma Chemical Co., St. Louis, MO, United States) was prepared by dissolving CHL in ethyl alcohol to a final concentration of 1.024 mg/mL. The MICs of CHL against *S. aureus* AB91118 and R1-3-1 were determined using a broth dilution method (Wiegand et al., 2008). Briefly, bacteria (10^5^ CFU/mL) were inoculated into LB medium with serial two-fold dilution CHL from concentration of 256 μg/mL. After incubation for 20 h at 37^o^C, minimum inhibitory concentration (MIC) was determined as the CHL concentration at which no visible growth of *S. aureus* was observed.

### Growth Rate of *S*. *aureus* R1-3-1

The antimicrobial activity of chloramphenicol alone, phage alone, and the combination of the two was evaluated against *S. aureus* R1-3-1 as described previously (Jo et al., 2016). R1-3-1 prepared in LB to a concentration of 10^6^ CFU/mL was incubated in a 96-well plate for 16 h in the presence of CHL (1/4 MIC or 1/2 MIC; 1 μg/mL or 2 μg/mL), LQ7 phage (10^6^ PFU/mL), or the combination (1 μg/mL or 2 μg/mL of CHL and 10^6^ PFU/mL of the phage). LB medium was used as a blank control and R1-3-1 without chloramphenicol and phage was used as a growth control. The optical density of the wells at 600 nm (OD_600_) was tracked using a microplate reader (Biotek, United States). The experiments were performed in triplicate.

### Mutant Rates Assay

The mutant rates of *S. aureus* AB91118 resistant to CHL, LQ7 or ELQ7P-20 were estimated, in triplicate, according to the method described previously (Jo et al., 2016; Valério et al., 2017). *S. aureus* AB91118 (5 × 10^6^ CFU/mL) was cultured in presence of CHL (at the concentration of 1/2 MIC), LQ7 (5 × 10^6^ PFU/mL), ELQ7P-20 (5 × 10^6^ PFU/mL), or different combinations of the three for 48 h first. A control was set by culturing *S. aureus* AB91118 (5 × 10^6^ CFU/mL) alone simultaneously. After the incubation, the bacteria was collected by centrifuge, washed with phosphate buffered saline (PBS: 137 mM NaCl, 2.7 mM KCl, 10 mM Na_2_HPO_4_, 2 mM KH_2_PO_4_) once, and then resuspended in PBS. The concentration of the bacteria in the suspensions were determined by plating serial dilutions onto LB agar plates. Finally, the mutant rates of *S. aureus* AB91118 after the 48 h incubation were determined by adjusting the bacterial concentration to 1 × 10^5^ CFU/mL and culturing on LB agar plates for 16 h at 37^o^C with CHL at the concentration of 1 MIC and individual phages at MOI of 1,000, respectively. The numbers of surviving colonies on the LB with and without CHL or phages were counted as resistant mutant numbers and total numbers, individually. Finally, the rates of mutants were calculated by dividing the number of mutants by the total number of bacteria.

## Results

### Isolation and Growth Rate of *S. aureus* Phage-Resistant Mutant

**FIGURE 2:**
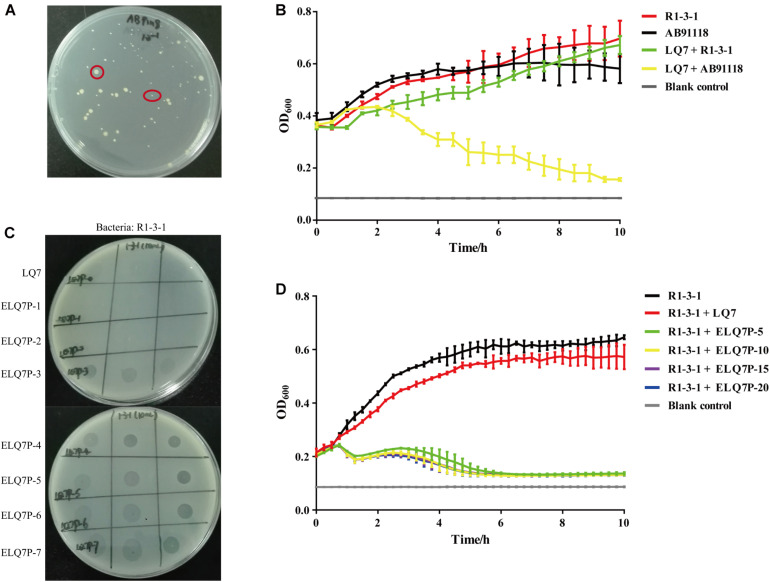
Isolation of *S. aureus* mutants resistant to lytic phage LQ7 and evolution of phage LQ7 against phage-resistant mutant R1-3-1. **(A)** Phage-resistant mutants on the double-layer plate show varied sizes after 24 h incubation of *S. aureus* AB91118 and LQ7. **(B)** Growth curves of R1-3-1 and AB91118 in presence of LQ7 at MOI = 10. Error bars show average values and SD of three independent experiments. **(C)** Spots formed on the double-layer plates by the evolved phages after different rounds of evolution against R1-3-1. **(D)** Growth curves of R1-3-1 in presence of LQ7 or evolve phages (ELQ7P-5, ELQ7P-10, ELQ7P-15, ELQ7P-20) at MOI = 1. Error bars show average values and SD of three independent experiments.

After incubation of *S. aureus* AB91118 and the lytic phage LQ7 on the double-layer plate for more than 24 h, some colonies were formed with varied sizes as shown in Figure 2A. These colonies are phage-resistant, which could not be killed by LQ7. After five repeats of incubation of a selected single resistant colony with phage LQ7, a resistant mutant was isolated and named as R1-3-1.

Although R1-3-1 showed resistance to LQ7, the presence of LQ7 at MOI of 10 could retard the growth rate of R1-3-1 compared with that of R1-3-1 without LQ7 (Figure 2B). Moreover, in the first one hour after mixing LQ7 and R1-3-1, the OD_600_ of the bacteria suspension did not increase, which indicated that R1-3-1 did not grow or grew very slowly in the first one hour.

Comparing the growth curve of AB91118 with that of R1-3-1, it could find that after 6 h the growth rate of R1-3-1 was significantly higher than that of AB91118. Even in the presence of LQ7, the growth rate of R1-3-1 from 8 to 10 h was higher than that of AB91118.

### Increased Killing Efficiency of the Evolved Phages on the Phage-Resistant Mutant

Experimental phage evolution using R1-3-1 as the host and LQ7 as the ancestral phage showed that after three rounds of evolution, the evolved phages started to show spots to R1-3-1 on the double-layer plates and the bactericidal activity of the evolved phages was retained for the subsequent rounds (Figure 2C). The growth curves in Figure 2D indicated that the evolved phages (ELQ7P-5, ELQ7P-10, ELQ7P-15, and ELQ7P-20) obtained the bactericidal activity to R1-3-1. The curves also showed that the initial bactericidal activity of the evolved phages was increased gradually as the evolution round increased. Moreover, consistent with the above results, the growth rate of R1-3-1 in the presence of LQ7 at MOI of 1 was lower than that of R1-3-1 in the absence of LQ7.

### The Morphology of Phages

**FIGURE 3:**
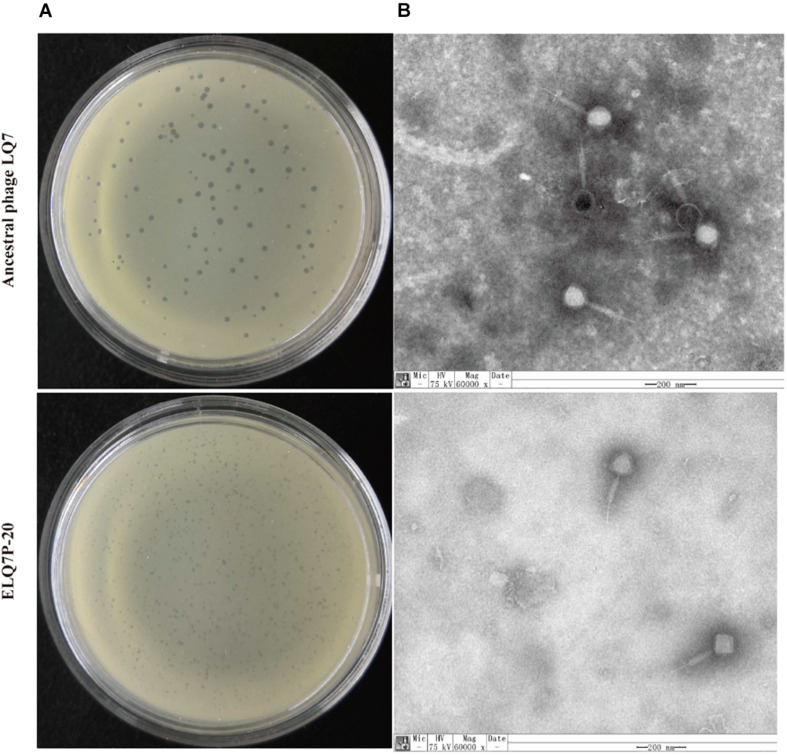
Plaques of LQ7 and ELQ7P-20 formed on lawns of AB91118 **(A)** and TEM images **(B)** of phage LQ7 and ELQ7P-20 stained negatively with freshly prepared 2% phosphor tungstic acid. Scale bar = 200 nm.

On the double-layer plates, the single plaque sizes of the ancestral phage LQ7 were approximately 1 mm in diameter on a lawn of *S. aureus* AB91118, and the single plaque size of the evolved phages (ELQ7P-20) were a little smaller than that of LQ7 (Figure 3A). TEM analysis revealed there was no obvious change in the phage morphology between LQ7 and ELQ7P-20, and they all belong to *Myoviridae* family (Figure 3B). Ancestral phage LQ7 has an icosahedral capsid (88 ± 3 nm, *n* = 5) with a long contractile tail (200 ± 10 nm, *n* = 5) and evolved phage population ELQ7P-20 has an icosahedral capsid (97 ± 8 nm, *n* = 5) with a long contractile tail (197 ± 10 nm, *n* = 5). Other three evolved phages (ELQ7P-5, ELQ7P-10, ELQ7P-15) are almost identical to that of ELQ7P-20 (Figures didn’t show).

### Enhanced Adsorption of the Evolved Phages to Phage-Resistant R1-3-1

**FIGURE 4:**
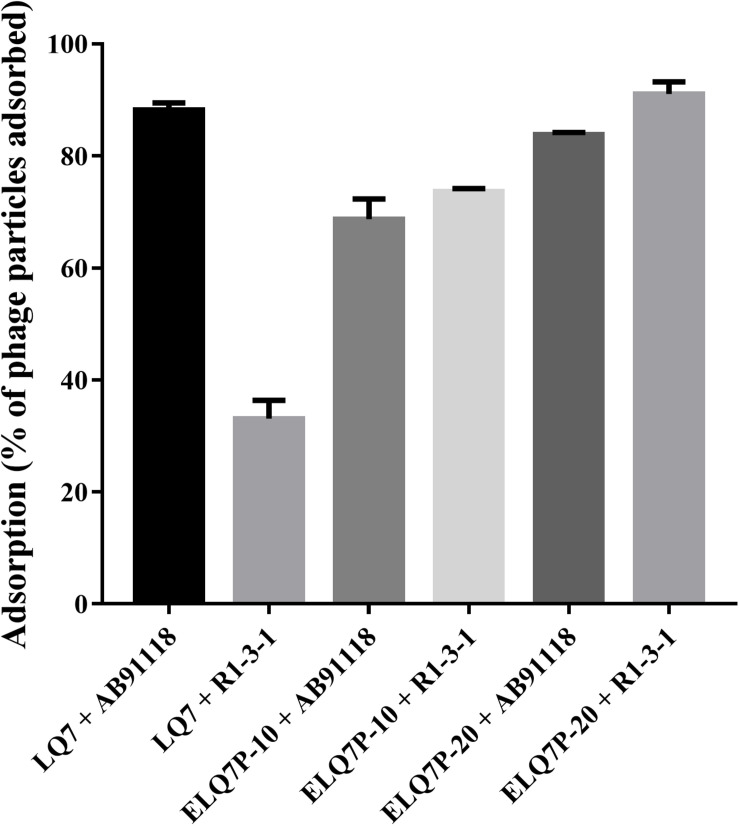
Adsorption rates (percentage of phage particles adsorbed) of evolved and ancestral phage against wild type *S. aureus* AB91118 and phage-resistance mutant *S. aureus* R1-3-1. Error bars show average values and SD of three independent experiments.

As shown in Figure 4, ancestral phage LQ7 demonstrated 88.2% adsorption rate to *S. aureus* AB91118 and 33.1% adsorption rate to *S. aureus* R1-3-1, suggesting the adsorption ability of LQ7 to phage-resistant R1-3-1 has decreased significantly. In comparison, the evolved phages ELQ7P-20 showed 83.8% adsorption rate to AB91118 and 91.0% adsorption rate to R1-3-1. The adsorption rates of the evolved phage ELQ7P-10 to both bacteria were between that of LQ7 and ELQ7P-20.

## Genome Analysis

### Mutations in the Genomes of Bacteria and Phages

Both genomes of *S. aureus* AB91118 and R1-3-1 were found having a size of 2,852,442 bp, including 2,654 genes, 19 rRNA, 61 tRNA, and 1 tmRNA. The average sequencing depths of *S. aureus* AB91118 and R1-3-1 were 1,516 and 1,239, respectively (Supplementary Figure 1A). There is only one mutation at site 2,48,520 (non-coding region) in the genome of R1-3-1, compared with that of AB91118.

The complete genome of the ancestral LQ7 is 138,579 bp in size containing 193 genes. The average sequencing depths of LQ7, ELQ7P-10 and ELQ7P-20 were 24,530, 25,737, and 23,930, respectively (Supplementary Figure 1B). Compared with the genome of LQ7, there are five mutations in the evolved phages ELQ7P-10 and ELQ7P-20 (Table 1). These mutations caused four amino acid non-synonymous substitutions.

**TABLE 1:** Mutations in the evolved phage populations.

Mutation site in the genome	CDs	Encoded protein	Protein length (aa)	Amino acid change (position)
54,927	LQ7_57	Capsid and scaffold protein (contain carbohydrate binding domain)	637	Asp → Asn (577)
91,133				
91,137				
91,140	LQ7_103	Sigma factor	219	Asn → Tyr (93) Ser → Val (94)
91,141				

### Analysis of Polymorphism Sites in the Genomes of Bacteria and Bacteriophage

Although the genomes of the bacteria and the phage LQ7 were determined by extracting genomic DNA from bacteria and phages growing from single colony of the bacteria and the phage, respectively, there were some polymorphism sites found to exist in the genomes, i.e., there are alternative bases with minor frequencies at certain sites of the genomes. The detailed four base frequency information on polymorphism sites in the genomes of the bacteria and the phages are listed in the Supplementary Table 1. Existence of some of the polymorphism sites with high MAF were verified by PCR-Sanger sequencing as shown in Supplementary Figure 3.

**FIGURE 5:**
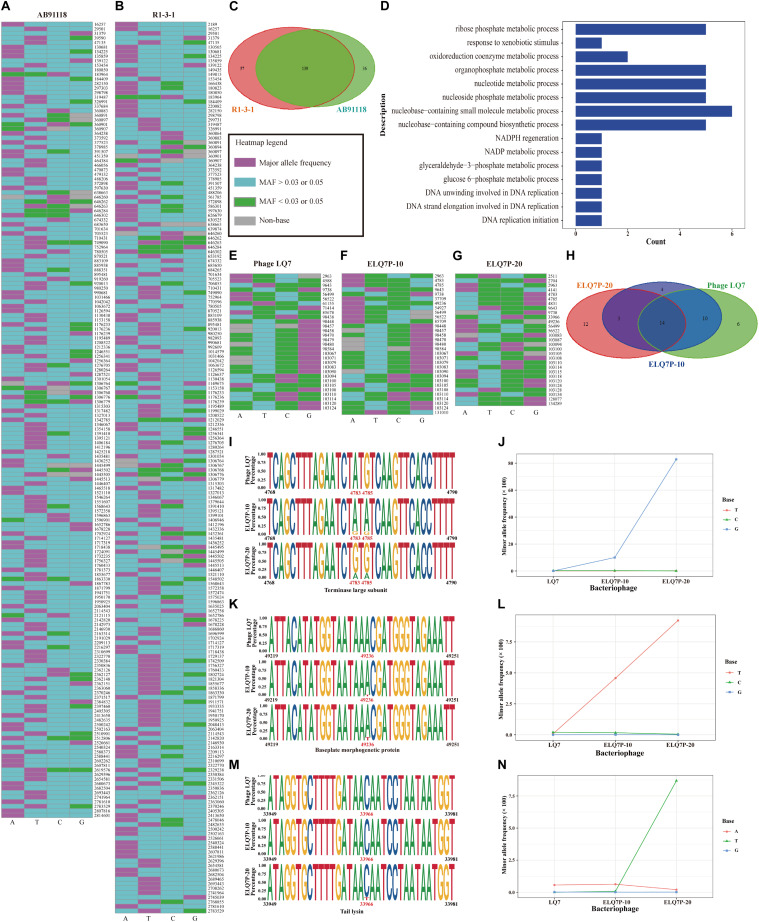
Genetic polymorphism sites in the genomes of bacteria and phages. **(A)** Polymorphisms heatmap of bacteria AB91118. **(B)** Polymorphisms heatmap of bacteria R1-3-1. **(C)** Venn diagram of polymorphism sites in the genomes of bacteria. **(D)** Fifteen of fifty-nine enriched biological process based on the 38 genes with unique polymorphism sites in R1-3-1. **(E)** Heatmap of polymorphism sites in the genome of phage LQ7. **(F)** Heatmap of polymorphism sites in the genome of phage ELQ7P-10. **(G)** Heatmap of polymorphism sites in the genome of phage ELQ7P-20. **(H)** Venn diagram of polymorphism sites in the genomes of phages. **(I)** Polymorphism sites at position 4,783 and 4,785 in the terminase large subunit gene. **(J)** Frequency changes of 3 minor bases at the polymorphism site 4,783 in the genomes of the phages. **(K)** Polymorphism site at position 49,236 in the baseplate morphogenetic gene. **(L)** Frequency changes of 3 minor bases at the polymorphism site 49,236 in the genomes of the phages. **(M)** Polymorphism site at position 33,966 in the tail lysin gene. **(N)** Frequency changes of 3 minor bases at the polymorphism site 33,966 in the genomes of the phages.

Using the criteria of MAF larger than 0.05 (which is more than 7 times of the sequencing errors 0.58% for AB91118 and 0.72% for R1-3-1) in the sequencing reads for bacteria to identify the polymorphism, there were 175 and 196 nucleotide polymorphism sites found in the genomes of AB91118 and R1-3-1, respectively (Figures 5A,B). Most of these sites exist more than 3 different bases (Figures 5A,B). Among them, there were 139 polymorphism sites existing in the both genomes of AB91118 and R1-3-1 (Figure 5C). Based on the unique polymorphism sites in the 38 functional genes of R1-3-1, 59 biological processes were identified by gene ontology enrichment analysis (Figure 5D). Most of the biological processes are related to nucleotide metabolism, energy metabolism and biomass synthesis. In particular, a biological process that responded to xenobiotic stimuli was enriched. The complete information of GO enrichment was given in the Supplementary Table 2.

Polymorphism sites in the genomes of the phages were also identified. Using the criteria of MAF larger than 0.03, which is more than 9 times of the sequencing errors 0.35% for LQ7, 0.34% for ELQ7P-10 and 0.17% for ELQ7P-20, in the sequencing reads for the phages to identify the polymorphism, there were 30, 31, and 29 nucleotide polymorphism sites found in the genomes of LQ7, ELQ7P-10, and ELQ7P-20, respectively (Figures 5E–G). For the most of these sites, 2 different bases existed (Figures 5E–G). Among them, there were 14 polymorphism sites existing in all the three genomes of the phages (Figure 5H). The phage ELQ7P-10 has 7 polymorphism sites different from LQ7, while ELQ7P-20 has 15 polymorphism sites different from LQ7. From the Venn diagram shown in Figure 5H, ELQ7P-10 looks like a transition from LQ7 to ELQ7P-20. Particularly, there are three common polymorphism sites at position 4,783, 4,785, and 49,236 in the genomes of ELQ7P-10 and ELQ7P-20, which belong to two different CDs encoding terminase large subunit and baseplate morphogenetic protein, causing two non-synonymous amino acids change (terminase large subunit: M25V, baseplate morphogenetic protein: K83N). The detailed base frequencies of the polymorphism sites in the genomes of phages are listed in the Supplementary Table 3. These polymorphism sites mainly existed in the functional genes encoding terminase large subunit, baseplate morphogenetic protein and tail lysin (at position 33,966). Further analysis showed that it was almost base A at 4,783 position in the terminase large subunit gene in the ancestral phage LQ7, but the frequency of G base, which was very low in LQ7, increased by about 290 times in ELQ7P-10, and further increased by about eight times in ELQ7P-20 during the evolution process (Figures 5I,J). The other two polymorphism sites at position 49,236 and 33,966 showed similar base frequency changes during the evolution (Figures 5K–N).

## Effects of Bacteriostatic Antibiotics and Phage on Susceptibility of Bacteria to Phages and Antibiotics

Since the genome comparison between AB91118 and R1-3-1 revealed that there was only one mutation in the non-coding region, it was suspected that the polymorphism sites unique in R1-3-1 would contribute to the resistance of R1-3-1 to phage LQ7 also. Because the unique polymorphism sites in R1-3-1 mainly affect the biological processes related to nucleotide metabolism, energy metabolism and biomass synthesis (Figure 5D), chloramphenicol, an antibiotic inhibiting metabolism, was tested to see how it would affect susceptibility of the bacteria to the phage.

**FIGURE 6:**
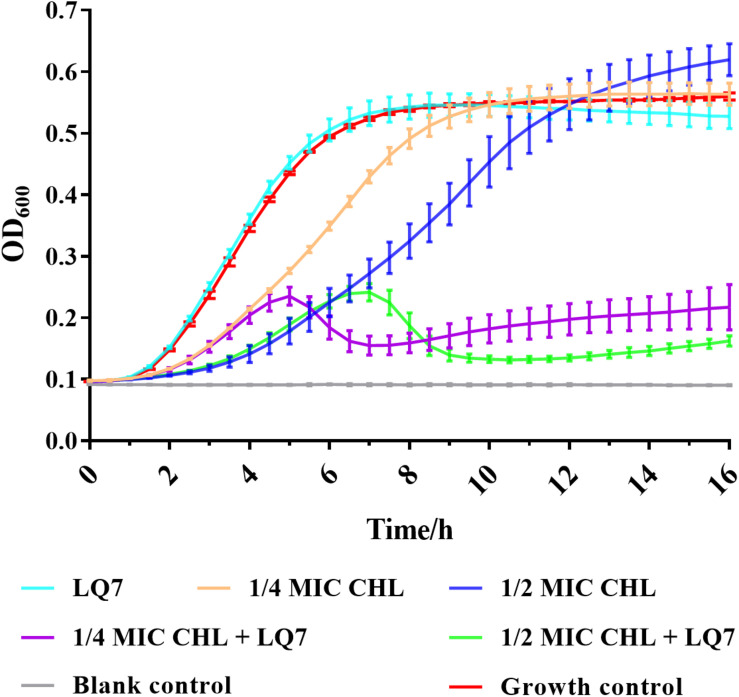
Effects of bacteriostatic antibiotic chloramphenicol and phage on the growth of R1-3-1.

The MIC of chloramphenicol was determined to be 4 μg/mL against both *S. aureus* AB91118 and mutant R1-3-1. From the growth curves in Figure 6, the growth of R1-3-1 could not be inhibited by LQ7 or chloramphenicol at 1/2 and 1/4 of MIC alone. However, the growth of R1-3-1 could be inhibited in the presence of both LQ7 and sublethal levels of chloramphenicol.

Further testing the growth of *S. aureus* AB91118 after 48 h incubation under different combinations of CHL and phages showed that AB91118 was inhibited most by the combination of CHL and ELQ7P-20, and followed by the combination of CHL and LQ7 (Table 2). At the same time, much less inhibition was observed by CHL alone or the phages alone. Comparing the phage groups without CHL, ELQ7P-20 showed better inhibition to AB91118 than LQ7. However, the combination of LQ7 and ELQ7P-20 did not improve the inhibition further.

**TABLE 2:** Changes of bacterial quantity and the rates of mutant resistant to CHL and phages after 48 h incubation with AB91118 under different treatments.

Treatment	Bacterial quantity after 48 h incubation	Mutant rates		
		Resistant to CHL (1 MIC)	Resistant to LQ7 (MOI 1000)	Resistant to ELQ7P-20 (MOI 1000)
CHL (1/2 MIC)	2.70 ± 0.99 × 10^9^	0.55 ± 0.03	4.50 ± 0.71 × 10^–5^	8.50 ± 0.71 × 10^–5^
LQ7 (MOI 1)	1.45 ± 0.21 × 10^9^	0.40 ± 0.11	6.10 ± 1.55 × 10^–4^	2.80 ± 1.41 × 10^–4^
ELQ7P-20 (MOI 1)	1.65 ± 1.52 × 10^8^	0.12 ± 0.07	8.70 ± 0.42 × 10^–3^	6.65 ± 0.35 × 10^–3^
CHL + LQ7	1.30 ± 0.42 × 10^6^	0.93 ± 0.01	2.25 ± 0.35 × 10^–2^	2.92 ± 1.29 × 10^–3^
CHL + ELQ7P-20	4.05 ± 1.70 × 10^5^	0.03 ± 0.01	5.96 ± 0.21 × 10^–3^	1.37 ± 0.11 × 10^–3^
LQ7 + ELQ7P-20	5.65 ± 1.62 × 10^8^	0.02 ± 0.01	8.50 ± 0.71 × 10^–2^	3.60 ± 0.57 × 10^–2^
CHL + LQ7 + ELQ7P-20	3.10 ± 1.27 × 10^6^	0.07 ± 0.01	3.75 ± 0.35 × 10^–3^	7.72 ± 0.71 × 10^–4^
Control	4.00 ± 0.01 × 10^9^	0	3.00 ± 1.41 × 10^–5^	7.00 ± 1.41 × 10^–5^

After the 48 h incubation, the bacterial resistance frequencies (the number of the mutant resistant to CHL, LQ7 or ELQ7P-20 over that of the total bacteria in the solution) were determined (Table 2). Generally, the bacterial resistance frequencies in all treatment groups became higher than that for the control. Treating the bacteria with CHL could significantly increase the resistance frequency to CHL, but not to the phages. While treating the bacteria with LQ7 or ELQ7P-20 alone could induce similar resistance frequencies to both phages, LQ7 treatment developed higher resistance frequency to CHL than the ELQ7P-20 treatment, and 10 folds lower frequencies to both phages than the ELQ7P-20 treatment. Treatments by combining any two of them showed that the combination of CHL and LQ7 developed the highest resistance frequency to CHL, and the combination of LQ7 and ELQ7P-20 induced the highest resistance frequencies to both phages and the lowest to CHL. Interestingly, the combination of CHL and ELQ7P-20 showed low resistance frequency to CHL and both phages. Further combining the three of them demonstrated slightly higher frequencies to CHL, but lower frequencies to both phages than the CHL and ELQ7P-20 combination.

## Discussion

It is quite common in lab to observe that there will be some resistant colonies shown after incubation of host bacterium and its lytic phage on agar plate for about 1 day. Because there exist only the host and the phage in the plate, it seems not possible for the host to obtain external genetic elements to obtain resistance. Therefore, there is an interesting question: how are the resistant colonies generated?

In the current study, by isolating a single colony from the agar plate after culturing the host with the ancestral phage LQ7, mutant R1-3-1 resistant to LQ7 could be obtained. Comparing the genome of R1-3-1 with that of AB91118, only one mutation at position 248,520 in the non-coding region could be identified, which is quite surprised if the resistance of R1-3-1 is exclusively due to this mutation. Fortunately, due to the enormous reads obtained during the genome sequencing, re-analyzing them revealed there exist many minor alleles in the genomes of both AB91118 and R1-3-1 (Figures 5A,B). The GO enrichment results showed that the polymorphism sites unique in the R1-3-1 genome distributed in thirty-eight functional genes, which were involved in biosynthesis, nucleotide metabolism and energy metabolism of the bacterium (Figure 5D). Coincidently the growth rate of R1-3-1 was found significantly higher than that of AB91118 (Figure 2B). Normally, high growth rates indicate high levels of biomass synthesis, which agrees with the GO enrichment results. These results reminded us that these unique polymorphism sites may contribute to the resistance of R1-3-1 to LQ7. More interestingly, R1-3-1 became sensitive to LQ7 in the presence of chloramphenicol at concentrations of 1/2 or 1/4 MIC (Figure 6). Previous studies have shown that bacteriostatic antibiotics chloramphenicol (CHL) could result in accumulation of amino acid, ATP, as well as NADH, due to reduced energy utilization and macromolecule biosynthesis of *S. aureus* (Lobritz et al., 2015; Stokes et al., 2019), which led us to try if CHL could be used to suppress the higher metabolic activity of R1-3-1 so that to reverse its resistance to the phage LQ7. As expected, the results in Figure 6 showed that nucleotide polymorphisms (minor alleles) that occurred in thirty-eight genes of R1-3-1 were not accidental and it is highly possible that the metabolic activity of R1-3-1 conferred partially its resistance to LQ7. It is worthy of note that there is a report having a similar postulate that the metabolic state of bacteria could influence their susceptibility to antibiotics (Stokes et al., 2019). Compare with other resistance mechanisms, the naturally existing minor alleles in the genome of the bacteria would provide the host a fast response to the selective pressure of the phage.

By the experimental evolution of LQ7 against R1-3-1, phages sensitive to R1-3-1 could be obtained as the plaques shown in Figure 3. Genome sequencing analysis showed that both mutation and the polymorphism of minor alleles would contribute to the susceptibility during the evolution process. Compared with the genome of LQ7, the evolved phage ELQ7P-10 and ELQ7P-20 showed five mutations causing three non-synonymous substitutions of amino acids (Table 1). Due to the capsid and scaffold protein in LQ7 containing the carbohydrate-binding domain, the mutation in this protein might contribute to the higher absorption rate of ELQ7P-10 to R1-3-1 (Figure 4). Previous studies reported that the widely existed carbohydrate-binding modules domain in phage can present in the tail protein to adsorb to its host (Dieterle et al., 2017; Hayes et al., 2018). At the same time, the polymorphism of minor alleles was also identified in the genomes of the ancestral phage LQ7 and the two evolved phages (Figures 5E–G). Based on the distribution of the minor alleles (Figure 5H), it was clearly shown that ELQ7P-10 was a transit between LQ7 and ELQ7P-20 during the evolution process. Specifically, there are six polymorphism sites associated with terminase large subunit gene, the baseplate morphogenetic gene and the tail lysin gene (Supplementary Table 3). The bacteriophage terminase consists of a small terminase subunit and a large terminase subunit, the latter possesses the ATPase and nuclease activities for packaging the DNA in the phage head, which is important in phage maturation (Weiditch et al., 2019). The increased proportion of non-synonymous polymorphism site on the baseplate morphogenetic protein (Figures 5K,L and Supplementary Figure 2B) might contribute to the higher adsorption capacity of ELQ7P-20 than that of ELQ7P-10 (Figure 4) since previous reports revealed that some phages in *Myoviridae* phage family evolved on baseplate and fibers genes associated with host recognition and adsorption (Akusobi et al., 2018; Habusha et al., 2019; Kizziah et al., 2020). Tail lysin participates in the digestion of bacterial cell walls and aids in the injection of phage DNA during infection Lavigne and Ceyssens (2012), but the polymorphism of minor allele occurred in this gene causes no amino acid change (Supplementary Figure 2C). Because none of the above polymorphism sites have been reported with the functions directly related to the metabolic activity of *S. aureus*, we believe other polymorphism sites in the genome of ELQ7P-10, which has 7 polymorphism sites different from LQ7, and ELQ7P-20, which has 15 polymorphism sites different from LQ7, might contribute its sensitivity to R1-3-1 also.

The arms race seems endless between phages and bacteria (Safari et al., 2020), which is true in the simplified system in the current study. As shown in Table 2, AB91118 was inhibited most by the combination of CHL and ELQ7P-20, and followed by the combination of CHL and LQ7 (Table 2). Therefore, it is beneficial to combine use of both CHL and phage to kill the bacteria. However, even with the combination of CHL and the phages, there are always some resistant mutants remained after the treatment. Actually, it could generally find the resistant mutant ratios became higher after the interaction of bacteria with either CHL or phages. It is quite interesting to find that treatments by combining CHL and LQ7 developed the highest mutant ratios resistant to CHL, but much smaller ratios resistant to CHL and both phages were observed after the treatment by the combination of CHL and ELQ7P-20. These results show that there are complementary effects between CHL and ELQ7P-20 to develop fewer mutants resistant to CHL and phages, but synergic effects between CHL and LQ7 to promote more mutants resistant to CHL and phages. Therefore, it may be case-by-case whether the combination of antibiotics and phages could produce less resistance while killing bacteria effectively (Valério et al., 2017). All these results hinted that it might be generally beneficial to combine antibiotic and phage for phage therapy, but complicated outcomes might be generated.

To be concluded, we found that the genetic polymorphism of minor alleles exists in the genomes of both bacteria and phages, which may be a new mechanism to enable host and phage get ready and have a fast response to the selective pressure by one to the other. Specifically, in the AB91118-LQ7 system studied here, it was found that the metabolic pathways involved in the genes with some unique polymorphic sites could be inhibited by CHL and make the resistant mutant R1-3-1 become sensitive to LQ7. Combination of CHL and ELQ7P-20 could produce less resistance and killing bacteria effectively. The polymorphism of minor alleles would open new clues to elucidate the interactions between bacteria and phages. But it needs further study to prove whether this is applicable to the complicated systems in nature, where host and phage exist with many other bacteria and phages.

## Data Availability Statement

The datasets presented in this study can be found in the Genome Warehouse in the National Genomics Data Center (National Genomics Data Center Members and Partners, 2020), Beijing Institute of Genomics (China National Center for Bioinformation), Chinese Academy of Sciences. The names of the repository/repositories and accession number(s) can be found below: https://bigd.big.ac.cn/gwh, GWHAOOZ00000000, https://bigd.big.ac.cn/gwh, GWHAOOY00000000, and https://bigd.big.ac.cn/gwh, CRA003040.

## Author Contributions

XZ designed and performed all experiments and wrote the manuscript. DX performed all the bioinformatics data analysis and wrote the manuscript. JY and HY attended the results discussion. PH performed the isolation of resistant R1-3-1. HW generated the idea, analyzed the results, and wrote the manuscript. All authors read and approved the final manuscript.

## Conflict of Interest

The authors declare that the research was conducted in the absence of any commercial or financial relationships that could be construed as a potential conflict of interest.

## Footnotes

1 http://www.vicbioinformatics.com/software.prokka.shtml

2 https://cran.r-project.org/bin/windows/base/old/3.5.3/

## Supplementary Material

Supplementary Figure 1 The sequencing depth of *Staphylococcus aureus* and phages.

Supplementary Figure 2 Changes in the amino acid sequence of terminal large subunit, baseplate morphogenetic protein and tail lysin caused by polymorphisms among phages.

Supplementary Figure 3 The evidence of Sanger sequencing data for five polymorphism sites.

Supplementary Table 1 Detailed four base frequency information on polymorphism sites in the genomes of the bacteria and the phages.

Supplementary Table 2 Fifty-nine biological processes identified by gene ontology enrichment analysis based on the unique polymorphism sites in the 38 functional genes of R1-3-1.

Supplementary Table 3 The protein information encoded by the gene with the polymorphic sites in the phage genomes and the numbers of four bases mapped to these sites.
