# Supplementary figures and images for: Genetic Polymorphism Drives Susceptibility Between Bacteria and Bacteriophages
Source: Front Microbiol. 2021 Mar 24;12:627897. doi: 10.3389/fmicb.2021.627897 (PMC8024471; doi:10.3389/fmicb.2021.627897)

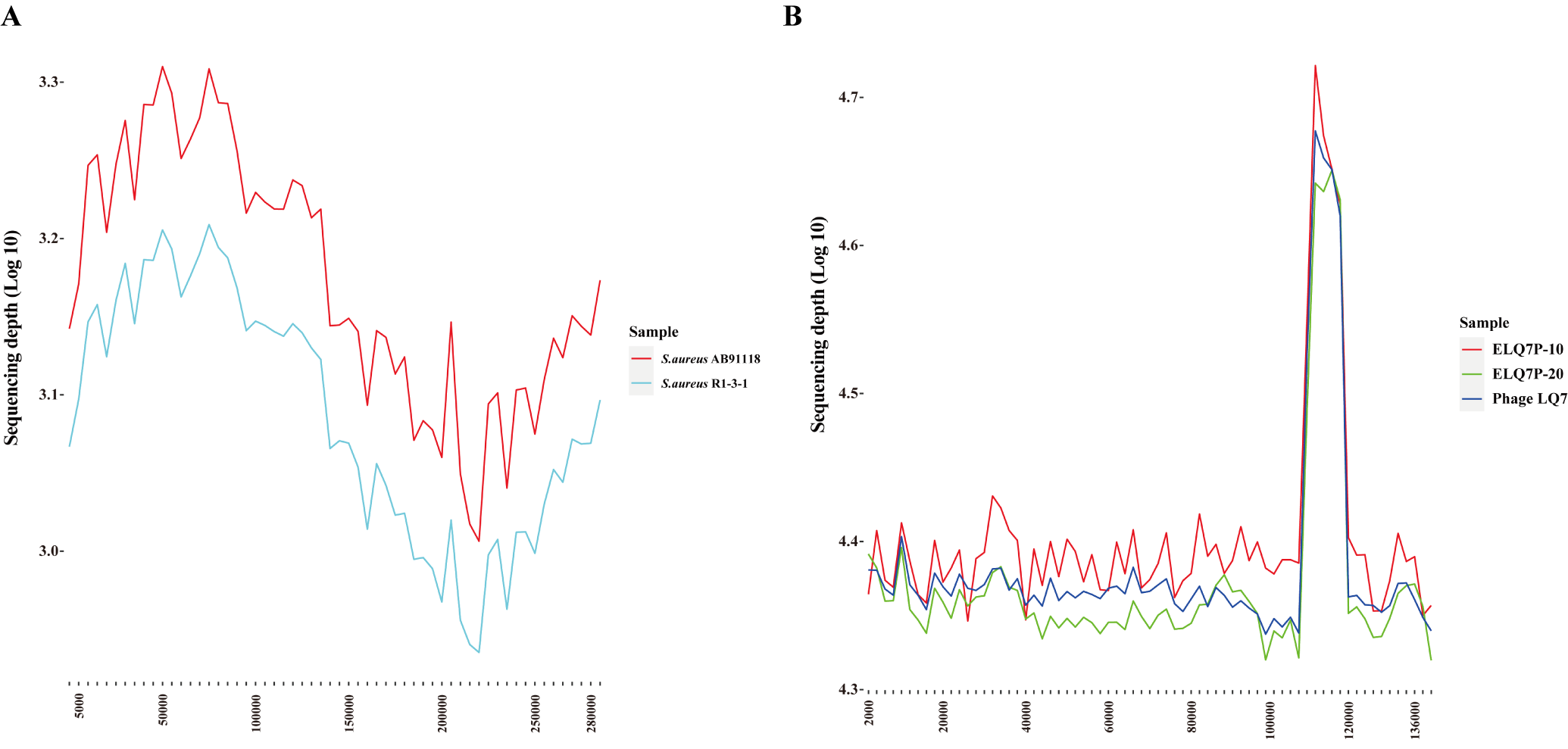

Supplement: Supplementary Figure 1 — The sequencing depth of Staphylococcus aureus and phages. [file Image_1.TIF]

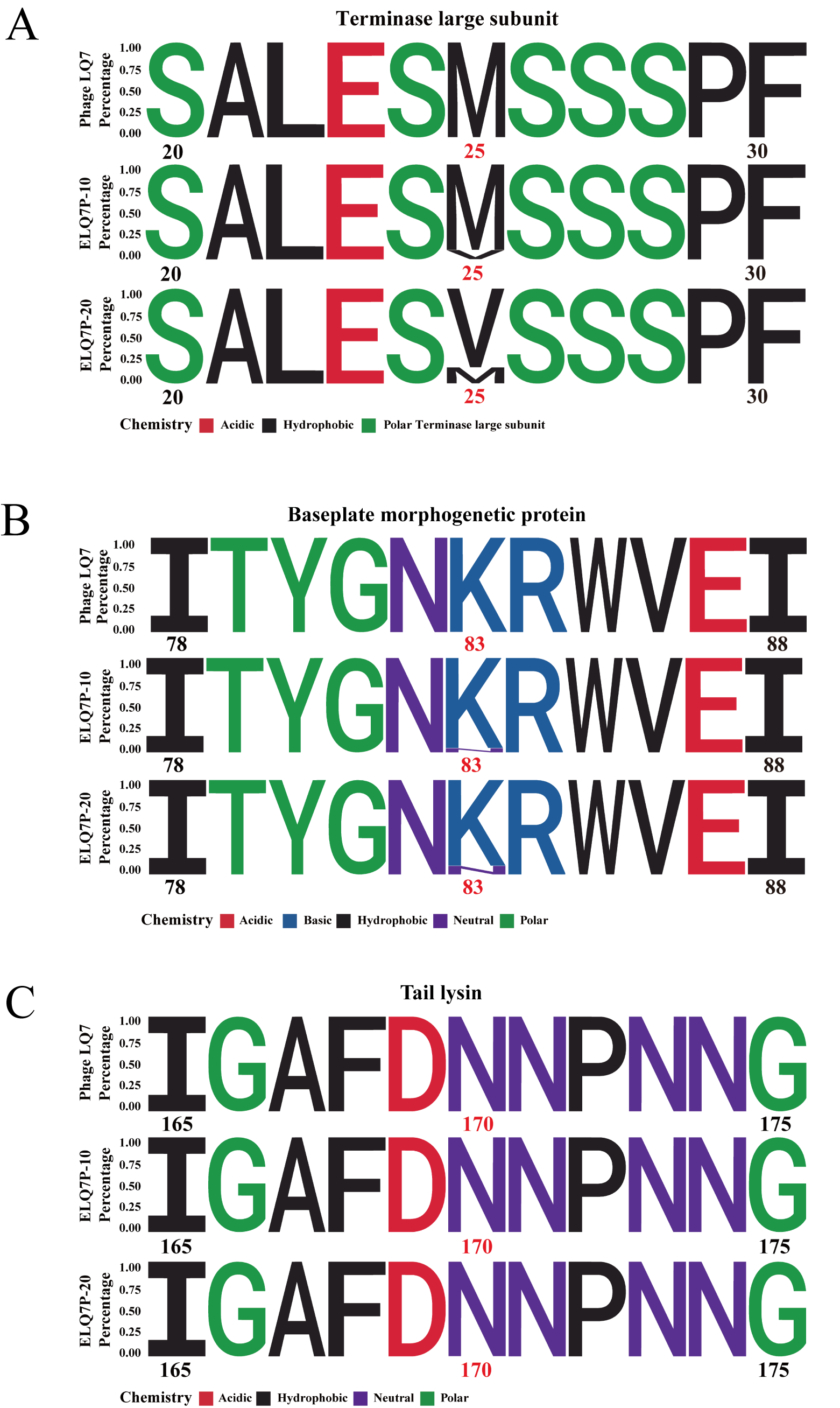

Supplement: Supplementary Figure 2 — Changes in the amino acid sequence of terminal large subunit, baseplate morphogenetic protein and tail lysin caused by polymorphisms among phages. [file Image_2.TIF]

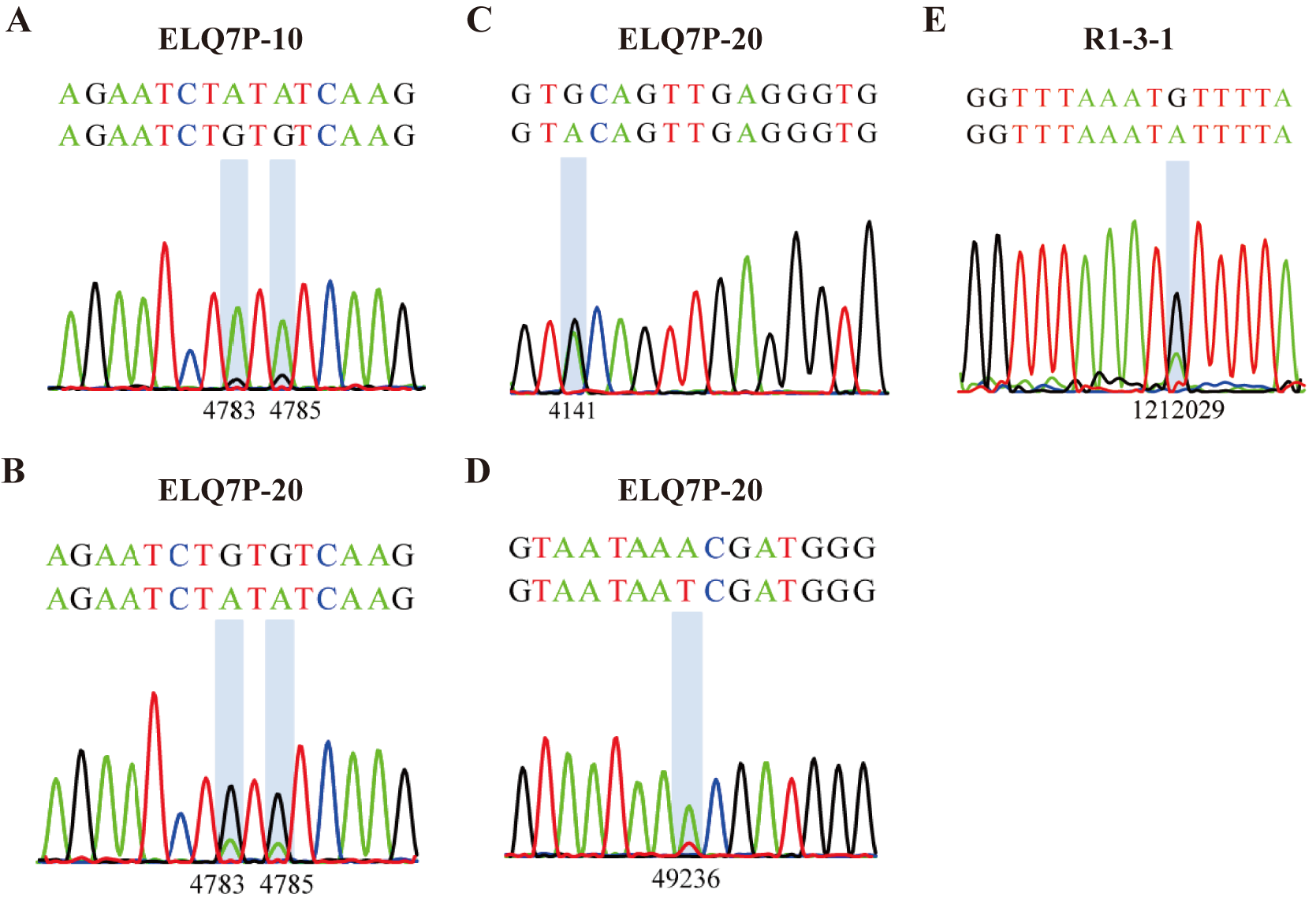

Supplement: Supplementary Figure 3 — The evidence of Sanger sequencing data for five polymorphism sites. [file Image_3.TIF]
